# Bioelectrical impedance analysis-guided fluid management promotes primary fascial closure after open abdomen: a randomized controlled trial

**DOI:** 10.1186/s40779-021-00329-0

**Published:** 2021-06-07

**Authors:** Kai Wang, Shi-Long Sun, Xin-Yu Wang, Cheng-Nan Chu, Ze-Hua Duan, Chao Yang, Bao-Chen Liu, Wei-Wei Ding, Wei-Qin Li, Jie-Shou Li

**Affiliations:** 1 Division of Trauma and Surgical Intensive Care Unit, Research Institute of General Surgery, Jinling Hospital, Medical School of Nanjing University, Nanjing 210002, No. 305 East Zhongshan Road, Nanjing, 210002 Jiangsu China; 2 Division of Trauma and Surgical Intensive Care Unit, Research Institute of General Surgery, The First School of Clinical Medicine, Southern Medical University, Nanjing, 210002 Jiangsu China

**Keywords:** Trauma, Open abdomen, Fluid overload, Fluid resuscitation, Primary fascial closure, Bioelectrical impedance analysis

## Abstract

**Background:**

Fluid overload (FO) after resuscitation is frequent and contributes to adverse outcomes among postinjury open abdomen (OA) patients. Bioelectrical impedance analysis (BIA) is a promising tool for monitoring fluid status and FO. Therefore, we sought to investigate the efficacy of BIA-directed fluid resuscitation among OA patients.

**Methods:**

A pragmatic, prospective, randomized, observer-blind, single-center trial was performed for all trauma patients requiring OA between January 2013 and December 2017 to a national referral center. A total of 140 postinjury OA patients were randomly assigned in a 1:1 ratio to receive either a BIA-directed fluid resuscitation (BIA) protocol that included fluid administration with monitoring of hemodynamic parameters and different degrees of interventions to achieve a negative fluid balance targeting the hydration level (HL) measured by BIA or a traditional fluid resuscitation (TRD) in which clinicians determined the fluid resuscitation regimen according to traditional parameters during 30 days of ICU management. The primary outcome was the 30-day primary fascial closure (PFC) rate. The secondary outcomes included the time to PFC, postoperative 7-day cumulative fluid balance (CFB) and adverse events within 30 days after OA. The Kaplan–Meier method and the log-rank test were utilized for PFC after OA. A generalized linear regression model for the time to PFC and CFB was built.

**Results:**

A total of 134 patients completed the trial (BIA, *n =* 66; TRD, *n =* 68). The BIA patients were significantly more likely to achieve PFC than the TRD patients (83.33% vs. 55.88%, *P <* 0.001). In the BIA group, the time to PFC occurred earlier than that of the TRD group by an average of 3.66 days (*P <* 0.001). Additionally, the BIA group showed a lower postoperative 7-day CFB by an average of 6632.80 ml (*P <* 0.001) and fewer complications.

**Conclusion:**

Among postinjury OA patients in the ICU, the use of BIA-guided fluid resuscitation resulted in a higher PFC rate and fewer severe complications than the traditional fluid resuscitation strategy.

**Supplementary Information:**

The online version contains supplementary material available at 10.1186/s40779-021-00329-0.

## Background

Open abdomen (OA) has been accepted as a therapeutic option for managing abdominal catastrophes over the last two decades [1–4]. However, OA patients have a high risk of developing various disastrous complications [5–7]. Promoting primary fascial closure (PFC) may reduce complications and improve outcomes [8, 9]. Historically, early aggressive crystalloid administration during intensive care unit (ICU) management resulted in fluid overload (FO) and continuing visceral edema [10, 11], which is a contributing factor of failure to achieve PFC [12–15]. Moreover, FO was linked to adverse outcomes in critically ill patients [16, 17]. Recently, crystalloid modulated and restrictive fluid resuscitation that avoids FO, including PRospective Observational Multicenter Major Trauma Transfusion (PROMMTT) study, balanced and damage control resuscitation, have been proved to achieve excellent results in trauma patients [18–21]. Reduction of resuscitation fluid volumes in OA patients was associated with an increased PFC rate during hypertonic saline, goal directed and direct peritoneal resuscitation [22–25]. Besides, our center also confirmed an association between FO and delayed PFC [26]. Therefore, judicious ICU fluid resuscitation therapy targeting assessment of FO to promote PFC is of great significance.

Traditionally, the FO calculation method was based on fluid balance measurements and body weight variations in critically ill patients. However, fluid balance studies using the difference in fluid inputs and outputs do not include insensible water loss and are less accurate [27, 28]. Moreover, it is difficult to measure body weight precisely in the ICU, and the value may change for reasons other than fluid infusion [29], which suggests the need for more convenient, reliable and accurate tools.

Bioelectrical impedance analysis (BIA) technology measures the total body, regional or segmental impedance of tissues and is derived from resistance (R) and reactance (Xc) by the transmission of electric currents at different frequencies [30, 31]. Rapid changes in hydration and soft tissue mass are identified by measurements standardized for height. This rapid, noninvasive, and convenient approach was reliable for ICU patients in whom the FO calculation was recorded using conventional methods [32]. The BIA technique appears to be valid, especially for assessment of FO by repeated measurements.

Therefore, we performed the trial to investigate the effects of fluid resuscitation protocols with adjustment determined according to the hydration level measured by BIA in compared with the traditional fluid resuscitation strategy determined by clinicians according to usual clinical parameters among OA patients. Our study hypothesis was that application of BIA to assess the hydration status among postinjury OA patients during ICU resuscitation could decrease fluid overload, promote PFC, and reduce complications caused by fluid overload.

## Methods

### Study design

**Fig. 1:**
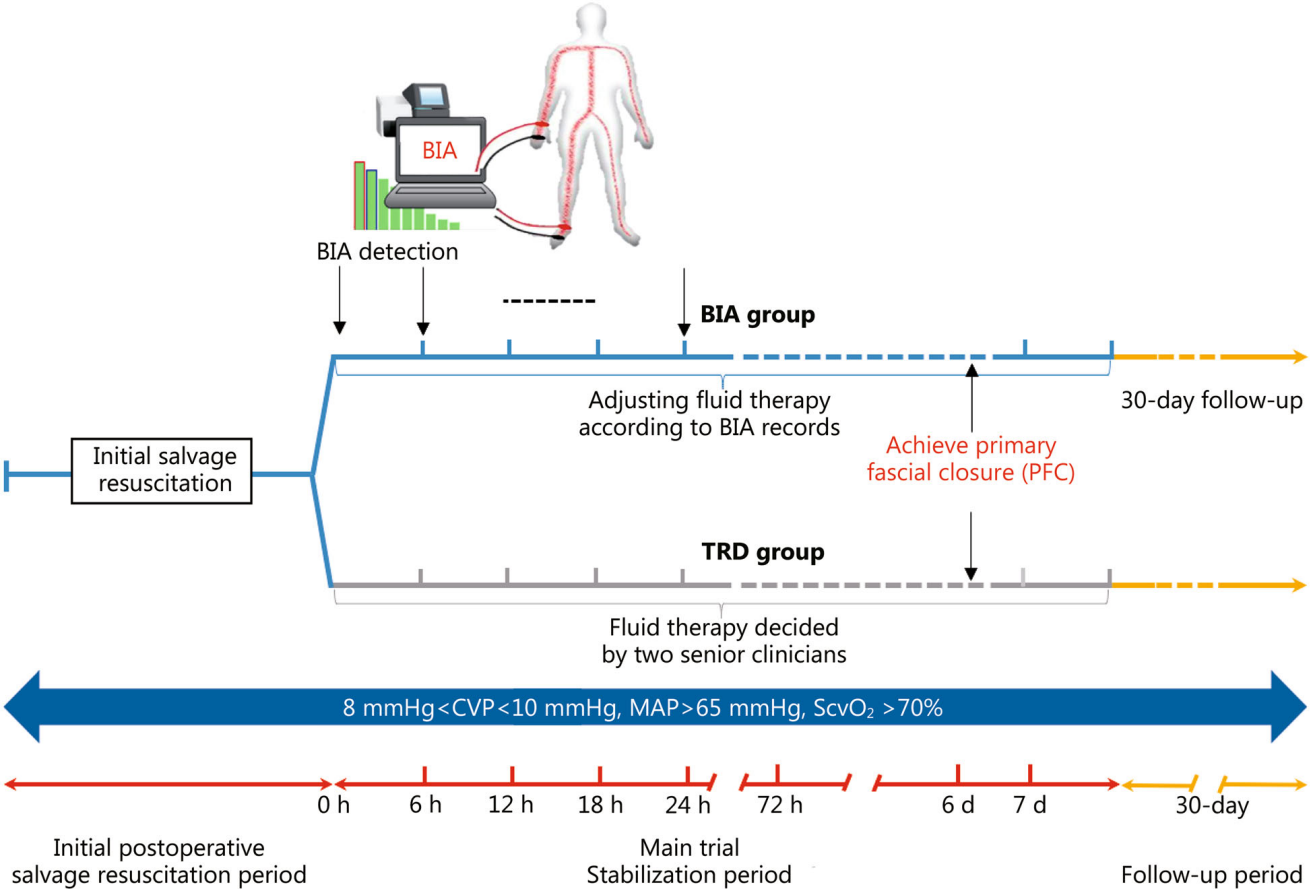
Trial design, the pragmatic, prospective, randomized, observer-blind, single-center trial had a duration of 7 days and a 30-day follow-up period. The patients were randomly assigned to two groups, and the different interventions were implemented accordingly. CVP central venous pressure, MAP mean arterial pressure, ScvO_2_ central venous oxygen saturation

The Bioelectrical impedance analysis-Guided Fluid Management (BGFM) was a pragmatic, prospective, randomized, observer-blind, single-center trial (clinicaltrials.gov Identifier: NCT03466684) conducted in two parallel groups (Fig. 1). This study was approved by the Institutional Ethics Committee of Jinling Hospital, Medical School of Nanjing University (No. 2012NZGKJ-096). Written informed consent was obtained from all participants or the closest relative of each participant after ICU admission. Patients were enrolled in the study between January 2013 and December 2017. The end of follow-up occurred in March 2018.

### Study setting

This study was set at the Research Institute of General Surgery of Jinling Hospital in Nanjing, Jiangsu, China. This national referral center has two Surgical Intensive Care Units (SICUs) that provide trauma and acute critical care surgery (ACCS) services for eastern China. After an abbreviated laparotomy, trauma patients with nonclosure of the abdominal fascia were admitted to the SICU.

### Study participants

Severely-injured adult patients admitted to SICU with OA who underwent emergent abbreviated laparotomy were considered eligible. The OA was defined as nonclosure of the abdominal fascia and skin after laparotomy that required temporary abdominal closure (TAC) [33]. The decision to perform OA was at the discretion of the attending physician and was made primarily for damage and abdominal contamination control, hemorrhage or planned re-laparotomy according to the damage control surgery (DCS) concept.

The TAC method was standardized among all available patients. TAC at the initial operation was performed with a vacuum-assisted and mesh-mediated fascial traction (VAWCM) as described previously [26, 34]. In brief, we placed a sterile perforated plastic sheet intra-abdominally to cover the viscera and sutured the oval-shaped polypropylene mesh (Prolene; Ethicon, Johnson & Johnson, Somerville, NJ) to the fascial edge with a running 0 monofilament suture. Besides, sterile gauze and moist laparotomy pads were placed to protect fascia and subcutaneous tissue and two silicone drain tubes were brought in caudally through the skin over the gauze. The drains were linked to a suction device with continuous topical negative pressure (100–150 mmHg). Next, the patients were taken back to the operating room every 2 days to achieve fascial closure. If possible, the abdominal wall was closed. Otherwise, we cut the mesh in the midline, changed the inner plastic sheet and gauze, and tightened the mesh by suturing it in the midline with a running 0 monofilament suture. The TAC was changed every 2 days. We decided to close the abdomen when the edge of the fascia is only 3–5 cm away with week tension assessed by pulling the fascial edges toward the midline. Finally, we removed the mesh, and closed the fascia, followed by skin closure. If the abdomen could not be closed after attempts within 15 days, then the abdomen was closed with biologics or skin grafting with a subsequent planned ventral hernia at the discretion of the senior clinicians [4, 35].

The exclusion criteria were as follows: a) age less than 18 years; b) pregnancy; c) lactation; d) limb amputations; e) mental disorders; f) diabetes mellitus; g) pre-existing blood disorders; h) pre-existing abdominal fistulas; i) pre-existing terminal illness; j) liver dysfunction (Child-Pugh class C); k) New York Heart Association (NYHA) class IV; l) chronic renal failure requiring dialysis; m) therapy with an extracorporeal membrane oxygenator (ECMO); n) enrolled in an ongoing, interventional randomized controlled trial (RCT); and o) expected to die within 24 h of ICU admission due to devastating injuries.

### Randomization

For patient allocation, a computer-generated randomization list gave an allocation table in permuted blocks (fixed block size, 4; allocation ratio, 1:1). Allocation concealment was conducted at the bedside using a sealed envelope to allow randomization by blinded administrative assistants immediately after the participant was deemed eligible.

### Study treatments

Eligible patients were randomly allocated to the traditional fluid resuscitation group (defined as TRD) or BIA-directed fluid resuscitation group (defined as BIA). After allocation, a multifrequency BIA with eight tactile electrodes (Inbody S10 Biospace, Biospace Co. Ltd., Seoul, Korea) was used to assess the body fluid status every 6 h within the first 72 h after admission to the ICU and daily for 4 days in both groups.

An alternating current of 250 mA at the frequency bands of 5, 50, 250, and 500 kHz was used, and two electrodes were built into every grip and plate as single-frequency BIA with eight electrodes. The patient’s skin was cleaned with saline before applying the electrodes to the arm and leg. The segmental impedance values in the arm, leg, and trunk were measured using a multifrequency analyzer for all frequencies. The hydration level (HL), fat mass (FM), body cell mass (BCM), internal cell water (ICW), external cell water (ECW), and regional fluid distribution were measured and automatically displayed. The HL and total body hydration status indices are evaluated by resistance and reactance through BIA. The migration of the vector in the nomogram results in variations of the numerical value of the HL. The OA patients were stratified into three hydration level categories (dehydrated, normally hydrated and hyperhydrated). According to the numerical scale for BIA, normal hydration was between 72.7 and 74.3%. Higher and lower values represented states of hyperhydration and dehydration, respectively. Hyperhydration was classified into three categories as follows: mild (74.3–81.0%), moderate (81.1–87.0%), and severe (> 87.0%) [36]. Similarly, dehydration was also classified into three categories: mild (71.1–72.7%), moderate (69.1–71.0%), and severe (≤ 69.0%) [16]. Trained nursing staff performed all anthropometric measurements.

First, all patients received aggressive hemodynamic support according to the standard protocol in our department during initial salvage resuscitation. Briefly, a 500-ml bolus of crystalloid was applied every 30 min to maintain central venous pressure (CVP) of 8 to 10 mmHg. If the mean arterial pressure (MAP) was below 65 mmHg, vasopressors were provided to maintain a MAP ≥65 mmHg. Red blood cells were transfused to promote ScvO_2_ when < 70%. The colloid used for fluid resuscitation was mainly blood products. If the patients had hypoproteinemia, albumin would be applied. Persistent anuria was assessed by senior clinicians, but we did not use the urine output to guide intravenous fluid resuscitation. When the patient CVP, MAP, and ScvO_2_ parameters met the above requirements, the patients were committed to the next stabilization period directed by the two fluid resuscitation strategies described below. The end point of resuscitation was postoperative day 7 in ICU after OA.

In the TRD group, the patients received a restricted intravenous fluid regimen or dehydration therapy based on the decision made by two senior clinicians according to the cumulative fluid balance recording and hemodynamic condition, such as the heart rate, blood pressure, CVP, MAP, urine output, cardiac echocardiography, lactate and body weight changes. BIA information was not available to the clinicians managing the patients in the TRD group, nor adjusted during fluid restriction or pharmacological and mechanical therapy.

**Fig. 2:**
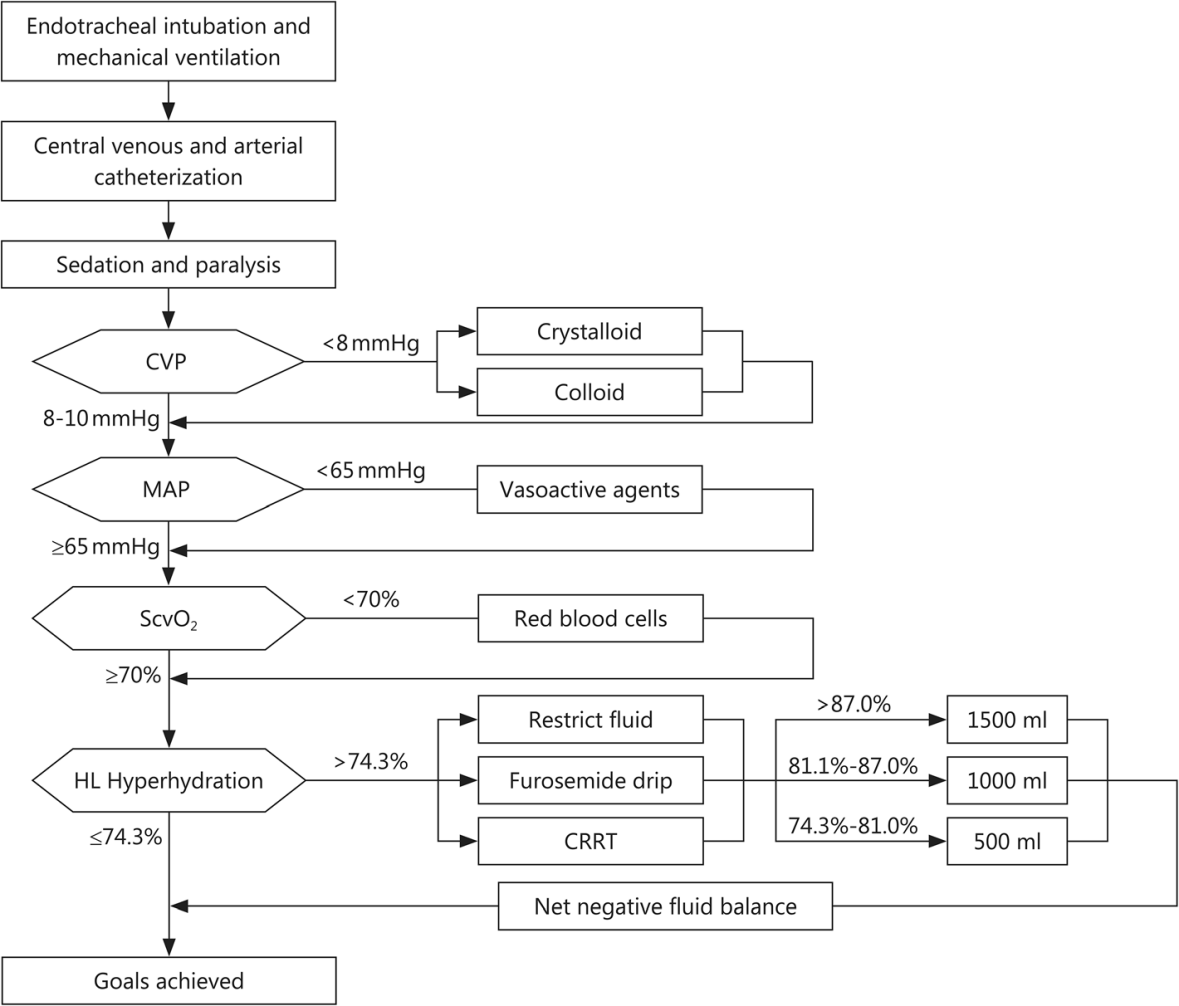
Protocol for BIA-directed fluid resuscitation. CVP central venous pressure, MAP mean arterial pressure, ScvO_2_ central venous oxygen saturation, HL hydration level, CRRT continuous renal replacement therapy

In contrast, in the BIA group, the protocol for patients who received BIA-directed resuscitation was as follows (Fig. 2). If hyperhydration (HL > 74.3%) was found after achieving the CVP, MAP, and ScvO_2_ goals, then the following fluid management strategy was applied for each 6-h period. If the HL was above 87.0% (severe level), fluid infusion was restricted, a furosemide drip was used, and continuous renal replacement therapy (CRRT) was initiated with an ultrafiltration rate when the patients were in failure or had an inadequate response to the above diuretic therapy that gave a net negative fluid balance of at least 1500 ml during the next 6 h. If the HL was 81.1–87.0% (moderate level), the above methods were used to trigger a net negative fluid balance (approximately 1000 ml) for the next six h. Similarly, if the HL was 74.3–81.0% (mild level), a net negative fluid balance of approximately 500 ml was achieved during the next 6 h of ICU hospitalization. If the HL was below 71.0% (i.e., a state of dehydration), the CVP, MAP, and ScvO_2_ were maintained as described above during ICU resuscitation. Vasoactive agents were used if necessary combined with close monitoring of the cardiovascular response, and a net negative fluid balance was abandoned during periods of hemodynamic instability.

### Blinding

Because implementation of the different fluid intervention regimens required clinician intervention, blinding of research clinicians participating in the protocol was inappropriate and infeasible. However, the other medical staff, sponsors, patients, and data and safety monitoring team remained unaware of the interventions during the trial.

### Data collection and outcomes measurements

Demographic and clinical characteristics were systematically recorded, including age, gender, mechanism of injury, indication for the open abdomen, intervention, laboratory tests, complications, and day of hospital and ICU admission. Patient populations were classified as blunt or penetrating according to the injury mechanism. The illness severity was based on the Acute Physiology and Chronic Health Evaluation (APACHE) II score, Sequential Organ Failure Assessment (SOFA) score, Abbreviated Injury Score (AIS) and Injury Severity Score (ISS) for trauma patients.

The fluid resuscitation volumes for the patients were systematically collected for postoperative 7 days. The total fluid intake was accurately monitored, which included administration of blood products, intravenous fluids, and drug infusions and provision of various nutrition support, such as enteral and parenteral nutrition. The fluid output included the CRRT output, urine output, tube drainage output, actual blood loss, nasogastric feeding tube output, stool output, insensible perspiration from both the skin and respiratory tract, and fluid drainage from TAC dressings. The cumulative sum of total blood product transfusions in the first 24 h, cumulative fluid balance and 24-h fluid balance over the first 7 days of OA management were recorded. During the first 72 h after OA, the vasopressors administered in the SICU were collected, including norepinephrine, vasopressin, phenylephrine, and dobutamine.

The primary outcome measured was the primary fascial closure rate (100% direct approximation of abdominal fascial edges) within 30 days. The secondary outcomes measured included days to PFC, postoperative 7-day cumulative fluid balance, 30-day mortality, hospital and ICU length of stay (LOS), length of mechanical ventilation and complications within 30 days after OA. All patients were followed 30 days from enrollment, and the outcomes at hospital discharge were included.

### Sample size

Postinjury OA patients with sIAI admitted to our referral center were anticipated to have a low fascial closure rate according to previous studies (almost 60%) and similar studies in other centers (33–60%) [26, 33, 37]. The fascial closure rate can exceed 80% with a 20 to 25% increase in intervention groups according to our previous pre-experiment trial. We estimated that an initial sample size of 120 would be required to detect a clinically significant 5% increase in the PCF rate at 80% power. The sample size was increased to 140 by the data and safety monitoring board according to an anticipated loss of 5 to 10% of patients entering the study due to changes in scheduled resuscitation.

### Statistical analysis

Continuous data were analyzed using Student’s t-test. Categorical data were analyzed using the Chi-square test or Fisher’s exact test whenever appropriate. The Kaplan–Meier method and the log-rank test were utilized for primary fascial closure up to 30 days after OA. Trends in the daily fluid input, daily fluid output, daily fluid balance, and cumulative fluid balance were illustrated using GraphPad Prism (version 7.0 for Mac, GraphPad Software, La Jolla, CA, USA) as the means and 95% confidence intervals (CIs) and compared using one-way ANOVA. A generalized linear regression model for the time until closure and cumulative fluid balance was built, including the covariates of BIA usage and any covariates with *P <* 0.2 in the statistical analyses. The data are reported as the means ± standard deviations (SDs) and medians (lower quartile, upper quartile) for continuous variables and as percentages for categorical variables. A *P* value of less than 0.05 was deemed statistically significant. The analyses were performed with the SAS statistical analysis software, version 9.3 (SAS Institute, Cary, NC).

## Results

**Fig. 3:**
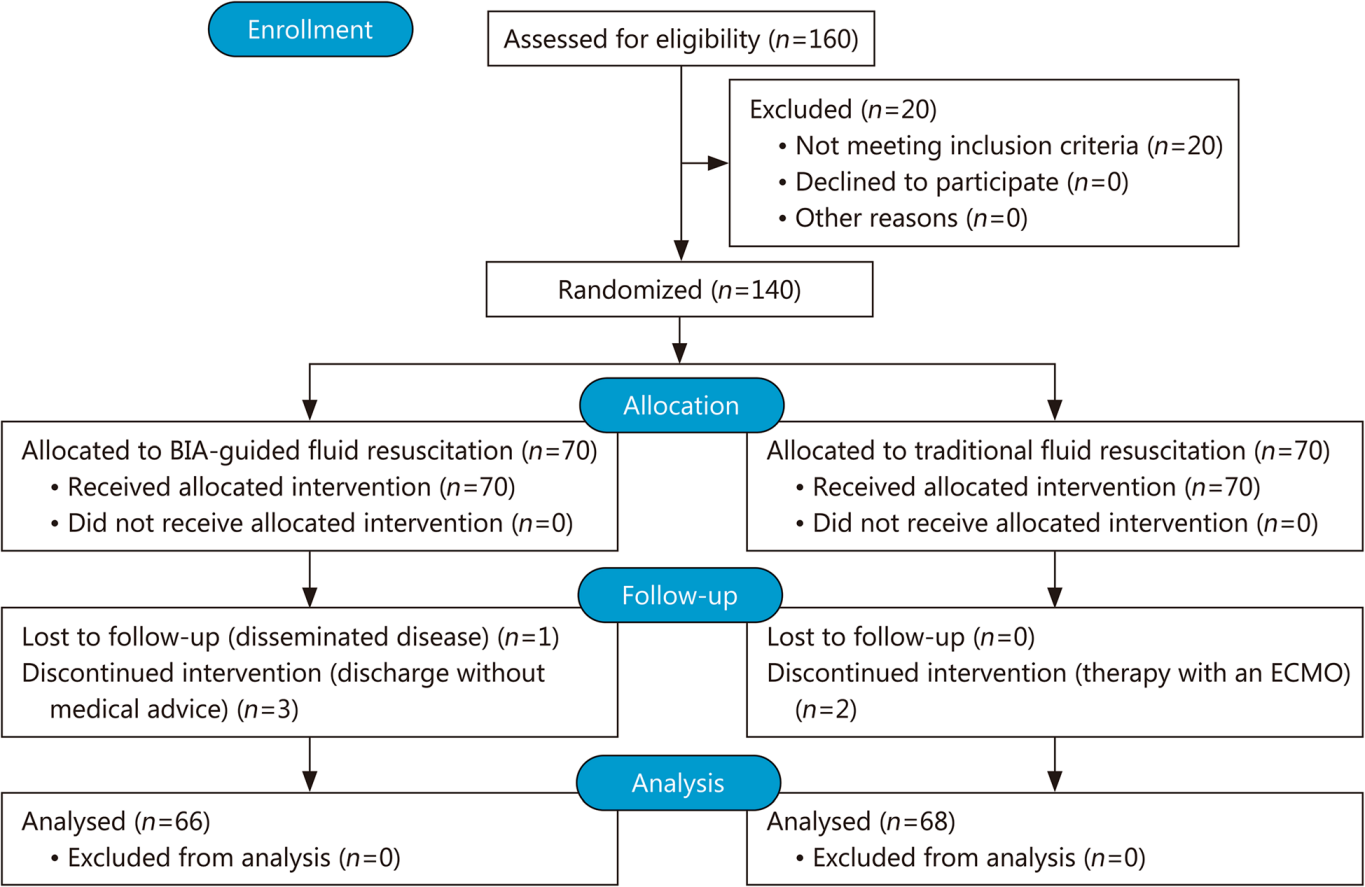
Patient CONSORT (Consolidated Standards of Reporting Trials) flow chart BIA bioelectrical impedance analysis, CONSORT Consolidated Standards of Reporting Trials, ECMO extracorporeal membrane oxygenator.

Between January 2013 and December 2017, a total of 160 patients undergoing OA with temporary abdominal closure were admitted to the two SICUs in our department. Of these patients, 20 were excluded (pre-existing abdominal fistulas, received prior therapy during ICU stay, age < 18 years, advanced liver dysfunction, limb amputations, chronic renal failure requiring dialysis, or therapy with an ECMO). A total of 140 participants were randomized (4 and 2 patients were lost to follow-up or discontinued the intervention in the BIA and TRD groups, respectively), and 134 participants were treated (66 in the BIA group and 68 in the TRD group; Fig. 3). The demographic characteristics, illness severity, and BIA data of the two clinical groups were shown in Table 1. The indications for OA were similar for the two groups.

**Table 1:** Patient characteristics and perioperative data by study group

Characteristic	BIA group (***n =*** 66)	TRD group (***n =*** 68)	***P*** value^*^
Age [years, mean ± SD]	46.60 ± 11.80	46.40 ± 7.70	0.904
Male, sex [*n* (%)]	39 (59.09)	39 (57.35)	0.838
BMI [kg/m^2^, mean ± SD]	24.00 ± 3.10	24.20 ± 2.40	0.663
Mechanism of injury [*n* (%)]			0.619
Blunt	48 (72.7)	52 (76.5)	
Penetrating	18 (27.3)	16 (23.5)	
AIS [mean ± SD]	8.20 ± 1.40	7.90 ± 1.50	0.335
ISS [mean ± SD]	24.80 ± 4.10	23.90 ± 3.60	0.208
APACHE II [mean ± SD]	24.00 ± 2.50	23.50 ± 2.60	0.267
SOFA [mean ± SD]	10.10 ± 2.30	9.80 ± 2.00	0.362
Indication for open abdomen [*n* (%)]			0.944
Damage control	29 (43.94)	30 (44.12)	
Intra-abdominal infections	28 (42.42)	30 (44.12)	
ACS	9 (13.64)	8 (11.76)	
Number of reoperations [*n,* mean ± SD]	1.65 ± 0.77	1.90 ± 0.88	0.090
Intraoperative fluid balance [ml, mean ± SD]	1480.60 ± 511.20	1394.90 ± 585.50	0.369
POD 1 blood products [U, mean ± SD]	10.05 ± 4.00	10.29 ± 3.90	0.714
Number of vasopressors [*n,* mean ± SD]	2.35 ± 0.97	2.38 ± 0.85	0.830
Hydration level on admission to ICU [%, mean ± SD]	79.60 ± 3.30	78.80 ± 5.00	0.233
POD 7 hydration level [%, mean ± SD]	71.50 ± 3.50	76.70 ± 3.70	< 0.001
Mean value of hydration level [%, mean ± SD]	74.60 ± 4.60	78.40 ± 4.50	< 0.001

*BIA* bioelectrical impedance analysis, *TRD* traditional fluid resuscitation, *BMI* body mass index, *AIS* abbreviated injury score, *ISS* injury severity score, *APACHE II* acute physiology and chronic health evaluation score II, *SOFA* sequential organ failure assessment, *ACS* acute compartment syndrome, *SD* standard deviation, *POD* postoperative day. ^*^P-values less than 0.05 were considered as statistically significant

### Fascial closure

**Fig. 4:**
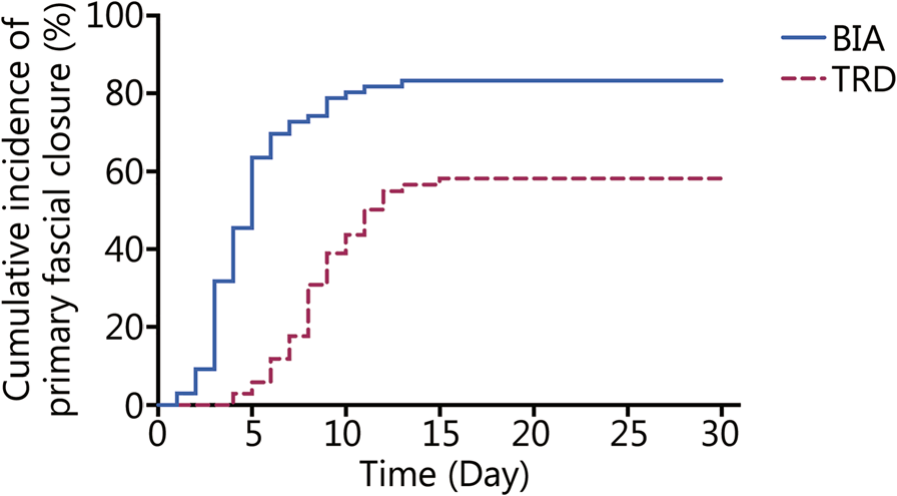
Cumulative incidence of primary fascial closure. Patients resuscitated by TRD (red, dotted line) were more likely to be delayed than those in the BIA group, who were more likely to be closed with a high rate (blue, full line). BIA bioelectrical impedance analysis, ECMO extracorporeal membrane oxygenator

All trauma patients with OA were taken back to the operating room for multiple abdominal operations before achieving fascial closure. The number of reoperations was similar between groups. The primary fascial closure rate was significantly higher in the BIA group than in the TRD group (83.33% vs. 55.88%, *P <* 0.001; Table 2). The median days to PFC were significantly lower in the BIA group than in the TRD group [(4.7 ± 2.5) days vs. (8.55 ± 2.5) days, *P <* 0.001]. The cumulative incidence of PFC up to 30 days in the TRD group was significantly more likely to be delayed than that in the BIA group, which was more likely to be closed with a higher rate (*P <* 0.001; Fig. 4).

**Table 2:** Postoperative fluid balance, complications and patient outcomes

Characteristic	BIA group (***n =*** 66)	TRD group (***n =*** 68)	***P*** value^*^
Primary fascial closure [*n* (%)]	55 (83.33)	38 (55.88)	< 0.001
Time to fascial closure [days, mean ± SD]	4.7 ± 2.5	8.6 ± 2.5	< 0.001
POD 7 cumulative fluid balance [ml, mean ± SD]	4620.7 ± 4532.0	11,448.6 ± 4749.1	< 0.001
Intra-abdominal complications [*n* (%)]			
Superficial infection	24 (36.36)	35 (51.47)	0.078
Intra-abdominal abscess/sepsis	39 (59.09)	45 (66.18)	0.397
Enteroatmospheric fistula	3 (4.55)	21 (30.88)	< 0.001
Extra-abdominal complications [*n* (%)]			
Pneumonia	20 (30.30)	39 (57.35)	0.002^*^
Sepsis	26 (39.39)	38 (55.88)	0.056
Acute renal failure	6 (9.09)	9 (13.24)	0.447
ALI	2 (3.03)	15 (22.06)	0.002
Outcomes			
Ventilator days [days, mean ± SD]	13.5 ± 7.9	20.8 ± 8.1	< 0.001
ICU LOS [days, mean ± SD]	19.7 ± 6.9	23.1 ± 7.6	0.006
Hospital LOS [days, mean ± SD]	23.9 ± 7.1	27.7 ± 3.1	< 0.001
30-day mortality [*n* (%)]	9 (13.64)	16 (23.53)	0.142

Data are expressed as the mean ± SD for continuous variables, or percentage for categorical variables. *BIA* bioelectrical impedance analysis, *TRD* traditional fluid resuscitation, *ALI* acute lung injury, *ICU* intensive care unit, *LOS* length of stay, *POD* postoperative day, *SD* standard deviation. ^*^P-values less than 0.05 were considered as statistically significant

After generalized linear regression analysis of the time to fascial closure was conducted to adjust for the admission diagnosis and prognostic factors (age, male sex, BMI, illness severity, indications, number of reoperations, number of vasopressors and intraoperative fluid volume), we found that resuscitation guided by BIA reduced the time to PFC by an average of 3.66 days (Table 3).

**Table 3:** Generalized linear regression analysis of time to fascial closure, and cumulative fluid balance

Group	Time to fascial closure		*P*	Cumulative fluid balance		*P*
	βcoefficient	Standard error		βcoefficient	Standard error	
BIA vs. TRD	−3.66	0.49	< 0.001	−6632.80	746.91	< 0.001

Adjusted for age, male, BMI, severity of illness (mechanism of injury, AIS, ISS, APACHE II and SOFA score), indications, number of reoperations, number of vasopressors and intraoperative fluid volume. *BIA* bioelectrical impedance analysis, *TRD* traditional fluid resuscitation. *P*-values less than 0.05 were considered as statistically significant

### Fluid resuscitation

**Fig. 5:**
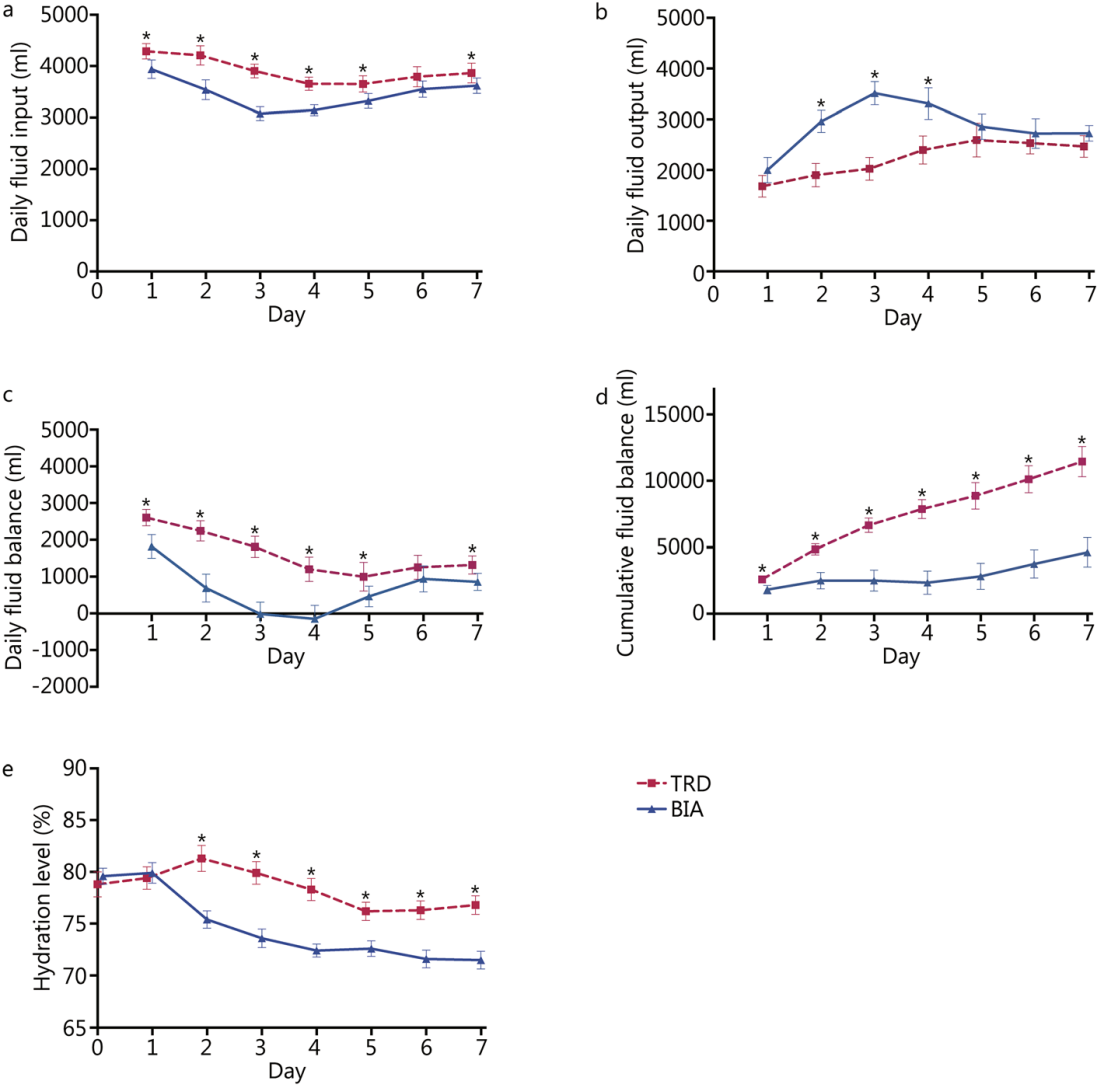
Time course of fluid resuscitation variables. a Daily fluid input. b Daily fluid output. c Daily fluid balance. d Cumulative fluid balance. e Hydration level. Means and 95% confidence intervals of pertinent variables for the first week after ICU admission. The fluid volume and bioelectrical impedance analysis variables of the BIA group are depicted with a blue full line, and those of the TRD group are depicted with a red dotted line. *P < 0.05, day-by-day pairwise comparison between the BIA and TRD groups. BIA bioelectrical impedance analysis, ECMO extracorporeal membrane oxygenator

Intraoperatively, the fluid input, fluid output, and total fluid balance did not differ between the groups (Table 1, Table S1). The cumulative sum of total blood product transfusions at postoperative day (POD) 1 were similar between the two groups. Over the first 7 days after OA, the amount of fluid input was lower in the BIA group than in the TRD group at postoperative days (PODs) 1–5 and 7 but did not significantly differ at POD 6 (Fig. 5a, Table S1). Compared with that of the TRD group, the BIA group underwent more fluid output at PODs 2–4 (Fig. 5b, Table S1). The amount of fluid output differed but failed to reach significance at PODs 1 and 5–7. Fluid resuscitation directed by BIA resulted in a significantly lower amount of daily fluid balance at PODs 1–5 and 7 (Fig. 5c, Table S1).

Over the 7 days of ICU resuscitation, the amount of cumulative fluid balance was lower in the BIA group at PODs 1–7 (Fig. 5d, Table 2, Table S1). Besides, HL was lower in the BIA group at PODs 2–7 (Fig. 5e, Table 1). Generalized linear regression for the postoperative 7-day CFB performed with the admission diagnosis and prognostic factors (age, male sex, BMI, illness severity, number of reoperations, number of vasopressors and intraoperative fluid volume) showed that the BIA group had a lower postoperative 7-day CFB by an average of 6632.80 ml (Table 3). More than two vasopressors were used per patient during ICU resuscitation, and the vasopressor requirements were similar between the two groups (*P* = 0.83; Table 1).

### Outcomes and complications

Complications were not infrequent in the two groups (Table 2). An enteroatmospheric fistula (EAF) was significantly more likely to occur in patients undergoing TRD (30.88% vs. 4.55%, *P <* 0.001). Among the extra-abdominal complications, patients resuscitated by TRD were also significantly more likely to have pneumonia (57.35% vs. 30.30%, *P* = 0.002) and an acute lung injury (ALI) (22.06% vs. 3.03%, *P <* 0.001). Compared with the patients in the TRD group, their BIA counterparts had fewer ventilator days [(13.5 ± 7.9) days vs. (20.8 ± 8.1) days, *P <* 0.001], shorter ICU lengths of stay [(19.7 ± 6.9) days vs. (23.1 ± 7.6) days, *P* = 0.007] and a reduced hospital length of stay [(23.9 ± 7.1) days vs. (27.7 ± 3.1) days, *P <* 0.001]. No significant difference was found in 30-day mortality between the BIA and TRD groups.

## Discussion

Currently, the fluid resuscitation strategy among postinjury OA patients in the ICU is predominantly guided by traditional protocols. Several retrospective studies of fluid resuscitation therapy have been implemented to achieve PFC and better outcomes among OA patients [20, 23]. However, no RCTs have supported the efficacy of rational fluid therapy for the prevention of FO and promotion of PFC in OA patients. To the best of our knowledge, this was the first randomized trial to use a multifrequency BIA device in an ICU setting to guide fluid resuscitation with a simplified protocol. The principal finding of the BGFM trial was that among ICU patients with OA, a postoperative fluid resuscitation regimen with adjustment determined by BIA resulted in a higher PFC rate, decreased FO, and lower complication rate than a traditional resuscitation strategy according to the usual clinical parameters.

Optimizing postoperative fluid therapy to promote PFC by alleviating fluid overload remains a challenge. Frequent development of FO in OA patients cannot be detected on time and that the degree of hydration cannot be appropriately assessed with effective measures. However, BIA, as promising tools, can be used to measure the body composition and fluid volume with an electric current transmitted through the body and has been validated in patients [38–40]. Our SICU began using this technology in 2008 and confirmed that it was reliable and easy to use for bedside evaluation [41]. In the current study, BIA was examined as a safe and effective technology in postinjury OA patients during ICU resuscitation.

Reduced fluid overload is an advantage of resuscitation directed by BIA. As an instantaneous technique to measure changes in the body fluid status of ICU patients, BIA can reflect the fluid overload state earlier and bypass errors due to fluid balance accounting. Additionally, serial BIA measurements accurately quantify the degree of edema, which establishes a target value to guide optimized fluid therapy and treatment targets for diuretics or ultrafiltration for CRRT patients. With the intervention based on the BIA protocol, an adequate circulating blood volume could be established, and complications resulting from fluid removal might be prevented. In a nutshell, BIA technique improved detection of the fluid overload state and guided optimized therapy, thereby preventing fluid overload in OA patients during resuscitation.

Notably, the application of the BIA technique was associated with an increased primary fascial closure rate and earlier fascial closure. Goussous and colleagues reported that a smaller fluid balance over the first 10 days was associated with PFC success among nontrauma patients [15]. Recently, we reported that fluid volume overload negatively influenced fascial closure among OA patients [26]. For the first time in ICU resuscitation practice, we combined BIA technology with a judicious fluid resuscitation protocol. The improved PFC rate in the BIA group was speculated to reduce FO and avoid massive visceral edema compared with those of the TRD protocol based on fluid balance registration in the ICU. Some studies reported PFC rates of 70% or higher using VAWCM, however, the PFC rate of trauma patients with severe intra-abdominal infection (sIAI) was much lower than other studies (35% vs. 90%). Padalino and colleagues reported that the PFC rate was only 33–60% in OA patients with sIAI. Many of the patients admitted to our referral center were severely injured accompanying sIAI transferred from local hospitals, and therefore showed a lower rate of PFC in TRD group. In this trial, the application of the BIA technique in ICU resuscitation significantly promoted PFC, which presented an encouraging result.

In the present study, the use of BIA was associated with a decreased incidence of adverse events, particularly EAF. Large volume resuscitation was associated with an increased frequency of complications, such as fistula formation, longer time to recovery from intestinal edema, and higher mortality [7]. Miller et al. [42] reported that fascial closure within 8 days contributed to fewer complications and better outcomes. Other studies using dynamic indices, better temporary abdominal closure dressings, and adjunctive peritoneal resuscitation decreased the time to primary fascial closure and improved the outcomes [33, 43, 44]. More significant primary fascial closure and fewer severe complications were shown for patients with BIA-directed fluid therapy. EAF is a disastrous intra-abdominal complication [45, 46], whose formation prevents OA patients from achieving PFC. We hypothesize that this decrease of EAF formation in BIA group was related to the promoted PFC and subsequently decreased the incidence and time of visceral exposure.

This study has several limitations. First of all, weight-based methods to determine the fluid overload status were not studied in this trial. Moreover, obesity, the size of the abdominal incision, the effect of intra-abdominal pressure on CVP, and other accompanying conditions might be important factors that we failed to control. Furthermore, we did not compare BIA-guided fluid resuscitation with other restrictive fluid management, and future prospective studies should be implemented to solidify the clinical results. Besides, problems inherent in the nature of this single-center study limit the generalizability of the research results to a broader population. Therefore, multicenter trials with a broader spectrum, including the use of BIA technique in balanced resuscitation practices may be sufficient to answer more critical clinical questions.

## Conclusions

Among postinjury OA patients in the ICU, the application of fluid resuscitation protocols with adjustments determined according to hydration level measured by BIA compared with the traditional fluid resuscitation strategy determined by the treating clinicians according to the usual clinical parameters resulted in higher PFC rates and fewer severe complications. Thus, close monitoring of the hydration status with the BIA technique may be routinely used in postinjury OA patients.

## Supplementary Information

**Additional file 1: Table S1** Intraoperative and postoperative fluid volume and drug management. *BIA* bioelectrical impedance analysis, *TRD* traditional fluid resuscitation, *POD* postoperative day.

## Data Availability

The datasets used and analysed during the current study are available from the corresponding author on reasonable request.

## Abbreviations

ACS: Abdominal compartment syndrome
AIS: Abbreviated injury score
ALI: Acute lung injury
APACHE II: Acute physiology and chronic health evaluation II
BIA: Bioelectrical impedance analysis
BMI: Body mass index
CIs: Confidence intervals
CFB: Cumulative fluid balance
CRRT: Continuous renal replacement therapy
CVP: Central venous pressure
DCS: Damage control surgery
EAF: Enteroatmospheric fistula
ECMO: extracorporeal membrane oxygenator
EN: Enteral nutrition
FM: Fat mass
FO: Fluid overload
HL: Hydration level
ICU: Intensive care unit
ISS: Injury severity score
LOS: Length of stay
MAP: Mean arterial pressure
NYHA: New York heart association
OA: Open abdomen
PFC: Primary fascial closure
POD: Postoperative day
R: Resistance
RCT: Randomized controlled trial
SAS: Statistical analysis system
SD: Standard deviation
sIAI: Severe intra-abdominal infection
SICU: Surgical intensive care unit
SOFA: Sequential organ failure assessment
TAC: Temporary abdominal closure
TRD: Traditional fluid resuscitation
VAWCM: Vacuum-assisted and mesh-mediated fascial traction
Xc: Reactance
