# Supplementary material for: Bioelectrical impedance analysis-guided fluid management promotes primary fascial closure after open abdomen: a randomized controlled trial
Source: Mil Med Res. 2021 Jun 7;8:36. doi: 10.1186/s40779-021-00329-0 (PMC8180439; doi:10.1186/s40779-021-00329-0)
Supplement: Supplementary file 1 — Additional file 1: Table S1 Intraoperative and postoperative fluid volume and drug management. BIA bioelectrical impedance analysis, TRD traditional fluid resuscitation, POD postoperative day. [file 40779_2021_329_MOESM1_ESM.docx]

**Table S1** Intraoperative and postoperative fluid volume and drug management

| **Characteristic** | **BIA group (*n =* 66)** | **TRD group (*n =* 68)** | ***P* value** |
| --- | --- | --- | --- |
| *IntraoPerative* [ml, mean ± SD] |  |  |  |
| Fluid input | 2378.8 ± 831.0 | 2430.9 ± 1118.9 | 0.760 |
| Fluid output | 898.2 ±509.5 | 1036.0±1211.0 | 0.39 |
| Fluid balance | 1480.6 ± 511.2 | 1394.9 ± 585.5 | 0.369 |
| *PostoPerative* [ml, mean ± SD] |  |  |  |
| Daily fluid input |  |  |  |
| POD 1 | 3939.7 ± 737.3 | 4291.3 ± 623.4 | 0.003 |
| POD 2 | 3537.6 ± 782.6 | 4211.4 ± 775.5 | < 0.001 |
| POD 3 | 3078.7 ± 541.8 | 3904.2 ± 565.2 | < 0.001 |
| POD 4 | 3145.7 ± 427.0 | 3655.2 ± 543.8 | < 0.001 |
| POD 5 | 3322.0 ± 587.9 | 3652.0 ± 649.3 | 0.003 |
| POD 6 | 3552.7 ± 644.8 | 3792.0 ± 823.6 | 0.063 |
| POD 7 | 3617.0 ± 618.2 | 3863.2 ± 804.8 | 0.049 |
| Daily fluid output |  |  |  |
| POD 1 | 2003.9 ± 1018.3 | 1681.7 ± 885.3 | 0.053 |
| POD 2 | 2958.6 ± 882.2 | 1901.4 ± 947.7 | < 0.001 |
| POD 3 | 3514.4 ± 931.4 | 2025.3 ± 931.8 | < 0.001 |
| POD 4 | 3311.0 ± 1252.9 | 2398.4 ± 1136.3 | < 0.001 |
| POD 5 | 2857.7 ± 1022.9 | 2595.1 ± 1380.8 | 0.212 |
| POD 6 | 2723.1 ± 1186.2 | 2536.7 ± 891.8 | 0.305 |
| POD 7 | 2728.7 ± 636.7 | 2468.1 ± 886.4 | 0.052 |
| Daily fluid balance |  |  |  |
| POD 1 | 1821.2 ± 1324.1 | 2606.6 ± 927.0 | 0.0001 |
| POD 2 | 690.2 ± 1540.9 | 2243.8 ± 1144.1 | < 0.0001 |
| POD 3 | -11.4± 1323.6 | 1809.8 ± 1200.4 | < 0.001 |
| POD 4 | -147.9 ± 1502.3 | 1204.8 ± 1385.0 | < 0.001 |
| POD 5 | 464.4 ± 1131.4 | 1003.8 ± 1614.6 | 0.028 |
| POD 6 | 940.5 ± 1464.0 | 1255.2 ± 1360.9 | 0.2 |
| POD 7 | 863.6 ± 953.5 | 1324.5 ± 1017.1 | 0.008 |
| Cumulative fluid balance |  |  |  |
| POD 1 | 1821.2 ± 1324.1 | 2606.6 ± 927.0 | < 0.001 |
| POD 2 | 2511.4 ± 2537.4 | 4850.4 ± 1694.1 | < 0.001 |
| POD 3 | 2500.0 ± 3264.4 | 6660.2 ± 2283.2 | < 0.001 |
| POD 4 | 2352.2 ± 3555.4 | 7865.0 ± 2948.9 | < 0.001 |
| POD 5 | 2816.5 ± 3988.9 | 8868.8 ± 4092.2 | < 0.001 |
| POD 6 | 3757.1 ± 4318.7 | 10124.1 ± 4193.5 | < 0.001 |
| POD 7 | 4620.7 ± 4532.2 | 11448.6 ± 4749.1 | < 0.001 |
| Number of vasopressors [*n*, mean ± SD] | 2.35 ± 0.97 | 2.38 ± 0.85 | 0.83 |

*BIA* bioelectrical impedance analysis, *TRD* traditional fluid resuscitation, *POD* postoperative day.
